# Deviations from the balanced time perspective, cognitive fusion, and self-compassion in individuals with or without a depression diagnosis: different mean profiles but common links to depressive symptoms

**DOI:** 10.3389/fpsyg.2023.1290676

**Published:** 2024-01-05

**Authors:** Anna Pyszkowska, Elisabeth Åström, Michael Rönnlund

**Affiliations:** ^1^Institute of Psychology, Faculty of Social Sciences, University of Silesia of Katowice, Katowice, Poland; ^2^Department of Psychology, Faculty of Social Sciences, Umeå University, Umeå, Sweden

**Keywords:** time perspective (TP), deviations from a balanced time perspective, depression, cognitive fusion, self-compassion

## Abstract

**Introduction:**

Prior research indicates that depressive symptoms in unselected or sub-clinical samples are associated with time perspective biases, including a more negative view of the past and a more fatalistic attitude toward the present. In the current study, we compared time perspective profiles for a clinical sample, with a depression diagnosis with that of a control group. Additionally, we considered a measure known as deviations from the balanced time perspective (DBTP) that capture deviations across time frames, not considered in previous studies. A second obejctive was to test a model involving DPTP as a mediator of the links between cognitive fusion and self-compassion with depressive symptoms.

**Method:**

In total, 300 individuals participated in the study, 150 participants with a depression diagnosis and 150 without a depression diagnoses. All participants filled in questions regarding background variables together with Polish adaptations of ZTPI, CFQ, SCS-S, and DASS-21 using a web-survey.

**Results:**

The results showed significantly higher scores on Past Negative and Present Fatalistic in the clinical sample. In line with the hypothesis the clinical group also displayed elevated DBTP scores (*d* = 0.75), a difference that remained significant when current symptoms were adjusted for. The results of structural equation modeling moreover indicate a major role of cognitive fusion (which, as expected, was strongly associated with DBTP) in predicting symptom burden, regardless of the clinical/non-clinical distinction, but. Still, DBTP accounted for significant (unique) variance in depressive symptoms. By contrast, the inclusion of cognitive fusion and DBTP eliminated the association of self-compassion and depressive symptoms.

**Conclusion:**

Taken together, the results indicate that levels of DBTP/fusion for persons with depression diagnosis is present regardless of current symptom burden. Thus, DBTP could be regarded as a risk factor of developing depression. Prospective research designs are needed to further evaluate the associations of the main constructs in this study and the extent to which they are predictive of future diagnosis and changes in symptom level.

## Introduction

Depression is one of the most common mental disorders, with almost one in five people experiencing at least one depressive episode in their lifetime (Mahli and Mann, 2018). According to WHO, approximately 128 million people worldwide suffered depression in 2023 (Institute of Health Metrics and Evaluation, 2023). Apart from symptoms such as depressed mood most of the day, anhedonia, fatigue and markedly diminished executive functions (e.g., memory, focus), a core characteristic of depression is negatively biased information processing (American Psychiatric Association, 2013). It includes selective attention to certain (mainly negative) aspects of one’s experiences, rigid, biased thinking patterns (Wen et al., 2023), and hinders positive information processing (Kube and Glombiewski, 2021). Several scholars have additionally highlighted that information processing in depression involves a disruption of temporal focus (i.e., Beck, 2008; Nolen-Hoeksema et al., 2008; Roepke and Seligman, 2016; Åström et al., 2019a,b). From a cognitive perspective, depression has long been explained as being caused and maintained by persistent negative thought patterns about oneself, the world, and the future (e.g., Beck’s cognitive triad of depression; Beck, 2008). Negative views of the future or negative prospection have especially been highlighted as a central mechanism behind depression as it may sustain negative views about oneself and the environment (Roepke and Seligman, 2016). Rumination, a common component of depressive symptomatology and which entails repetitive negative thinking about the past (e.g., Nolen-Hoeksema et al., 2008), is another example of how depressive cognitions can be characterized from the viewpoint of a temporal dimension.

The focus of this study is on time perspective in depression as it is linked with both rigid thinking patterns (*cf.* Pyszkowska and Rönnlund, 2021) and depressive symptoms (Micillo et al., 2022). Time perspective encompasses an individual’s focus on the past, present and the future as well as the valence put on each time frame (e.g., Zimbardo and Boyd, 1999). Prior studies that examined the associations of different time perspective dimensions and depressive symptoms typically involved the Zimbardo Time Perspective Inventory (ZTPI; Zimbardo and Boyd, 1999). ZTPI measures time perspective according to five subscales, two that concern the past, two that involve the present and one scale that concerns the future. Past Positive reflects a war and nostalgic view of the past (e.g., *Familiar childhood sights, sounds, and smells often bring back a flood of wonderful memories*), whereas Past Negative reflects an aversive view toward the past (e.g., *I think about the bad things that have happened to me in the past*). Present Hedonistic captures a life-for the moment attitude toward the present with little concern for the future (e.g., *I feel that it’s more important to enjoy what you are doing than to get work done on time*). Whereas Present Fatalistic, reflects a view of the present characterized by an external locus of control (e.g., *My life path is controlled by forces I cannot influence*). Future, finally, involves a general future orientation including planning and striving for future rewards (e.g., *When I want to achieve something, I set goals and consider specific means for reaching those goals*). According to the theory underlying the ZTPI, the relative weight and valence an individual places on each time frame often develops into a relatively stable disposition. Time perspective is thus outlined as a trait-like construct.

So far, studies on time perspective and depression mainly focused on symptom levels in unselect and relatively healthy samples. For example, Anagnostopoulos and Griva (2012) examined a Greek version of the ZTPI in a sample of 337 university students, reporting that higher Past Negative as well as higher Present Fatalistic were associated with scores on the Center of Epidemiological Studies Depression Scale. By contrast, Past Positive showed a significant negative association with depressive symptoms. The same patterns of association of Past Negative and Present Fatalistic with depressive symptoms were observed in a study by Desmyter and de Raedt (2012) that involved community dwelling older adults (see also Åström et al., 2019a,b). The only study we know of that directly compared individuals with a depression diagnosis and non-depressed participants was a recent study by Lefèvre et al. (2019). This was a small study (45 patients and 43 controls) but much in line with the studies on depressive symptoms, the patient group scored higher on Past Negative and Present Fatalistic. In addition, the clinical groups exhibited a lower level of Present Hedonistic compared with the control group.

Whereas the former studies focused on individual ZTPI subscales current research trends within the time perspective literature have moved toward also considering the deviation from an optimal (or balanced) time perspective profile (Zimbardo and Boyd, 2008). These studies typically considered a measure referred to as Deviations from a Balanced Time Perspective (DBTP; Zhang et al., 2013). DBTP is computed as the sum of differences between the individuals scores on ZTPI and the optimal score profile for the ZTPI, taking all five subscales into account. A higher DBTP score consequently indicates that a person has a more rigid and negatively biased time perspective, which is believed to correspond with a poorer ability to flexibly with between time frames in an adaptive manner (Zimbardo and Boyd, 2008). Research has shown that DBTP is associated with a variety of mental health outcomes (for a review, see Stolarski et al., 2020).

Two studies that we know of McKay and Cole (2020, 2023) examined the association of DBTP and depressive symptoms, once more in rather healthy university samples. In the first study (McKay and Cole, 2020), Past Negative was significantly associated with score on the Hospital and Anxiety Depression scale (mean *r* = 0.30 across two subsamples). Past Positive showed a significant reversed association (*r* = −0.31) with depressive symptoms in one sample, but not in a second sample (*r* = −0.11 n.s). Unlike the studies reviewed above, no significant association was observed for Present Fatalistic in either sample (*r* = 0.11; *r* = 0.19). Perhaps more surprisingly, the DPTP-symptom association did not exceed those of the individual ZTPI subscales (*r* = 0.31 in sample 1 and *r* = 30 in sample 2). In the second study (McKay and Cole, 2023), by contrast, none of the individual ZTPI dimensions were significantly associated with HADS-d scores, but so were two versions of the DBTP score (*r* = 0.31–0.33). Given the modest associations and difference in outcome across the two studies, the usefulness of DBTP in predicting depressive symptoms remains unclear.

On a theoretical level, DBTP bears resemblance with cognitive fusion, a construct that has been suggested as highly relevant for the development and maintenance of mental distress, including depression (Bardeen and Fergus, 2016). More specifically, defined as “the tendency for behavior to be overly regulated and influenced by cognition” (Gillanders et al., 2014, p. 84), cognitive fusion similarly entails rigid information processing. Several studies demonstrated that cognitive fusion is related to depressive symptoms (e.g., Carvalho et al., 2019; Pinto-Gouveia et al., 2020; Donati et al., 2021), also indicating that cognitive fusion may act as a mediator of the relationship between depression and other aspects commonly associated with depression, such as shame (Dinis et al., 2015) and rumination (e.g., Cookson et al., 2020).

Being fused and unable to flexibly distance oneself from the negative content of one’s thinking, should thus be similar to being overly “stuck” in specific time frames (i.e., resulting in higher DBTP). Both cognitive fusion and DBTP stand in stark contrast to self-compassion, a construct defined as a non-judgmental, flexible, and understanding attitude toward one’s own suffering, failure, and feelings of inadequacy when they arise (Neff, 2003). In contrast to cognitive fusion and DBTP, self-compassion is seen as a protective factor against depression (Körner et al., 2015; Zhang et al., 2019). Empirical studies have further demonstrated moderate to strong correlations between self-compassion and psychological flexibility on the one hand (e.g., Pyszkowska and Rönnlund, 2021) and on the other, negative correlations with cognitive fusion (Böge et al., 2022) and DBTP (Pyszkowska and Rönnlund, 2021).

Taken together, DBTP and cognitive fusion may both be characteristic of depressive information processing, whereas self-compassion instead represents more adaptive information processing, protecting against depression. The interconnectedness between DBTP, cognitive fusion and self-compassion is evident in their definitions, although the relationships between all three concepts and their relative weight on depression have not yet been empirically tested.

Given the paucity of studies the aim of the study was to examine the role of time perspective in depression. A first objective was to compare time perspective profiles for individuals with a depression diagnosis with a control group, including a comparison of DBTP levels. The measurement of current depressive symptoms allowed for examining the hypothesis that individuals with a depression diagnosis show a persistent (chronic) bias in time perspective even when symptom level was adjusted for, something which might be expected if distorted time perspective is a precursor of depression. Based on prior studies of correlations of depressive symptoms in relatedly healthy or subclinical samples, we our hypotheses were furthermore that the sample with a depression diagnosis would show significantly higher levels of negative TPs (Past Negative and Present Fatalistic, *cf.* McKay and Cole, 2023) compared to the controlled. Additionally, based on results of prior studies, we expected the depression group to show elevated levels of cognitive fusion (Gillanders et al., 2014), and lower self-compassion (Krieger et al., 2013).

A second objective was to examine the relationship between DBTP, cognitive fusion, self-compassion, and depressive symptoms. More specifically, we set out to test a hypothetical model in which the relationship between cognitive fusion and depression was mediated by DBTP (*cf.* Pyszkowska and Rönnlund, 2021). In line with previous research regarding cognitive rigidity, it was hypothesized that, regardless of depression diagnosis, cognitive fusion and DBTP would be associated with enhance levels of depressive symptoms (Ranjbar et al., 2022), and, together, diminish the relevance of self-compassion (Pyszkowska et al., 2021) in predicting depressive symptoms.

## Methods

### Participants

Participants were recruited through the DRB Polonia research panel using CAWI (computer-assisted web interviewing) methodology. Respondents were granted points that could be exchanged for gift cards for providing responses. All participants provided informed consent.

The inclusion criteria for a clinical sample were: (1) declaring a major depressive disorder diagnosis without psychotic symptoms at the time (former F32 in ICD-10, 6A70-6A7Z in ICD-11), (2) age over 18 years. For a nonclinical sample, they were (1) declaring not having a major depressive disorder diagnosis (or any other psychiatric diagnosis) at the time, (2) age over 18 years.

In total, 300 individuals participated in the study. Of these, 150 persons (106 women and 44 men) fulfilled diagnostic criteria for major depressive disorder and 36 persons (24%) reported a comorbid diagnosis, e.g., anxiety disorder (18 persons), bipolar disorder (6 persons), or borderline personality disorder (4 persons). Most of the participants (88%) had received treatment for depression (*n* = 36 for psychotherapy, *n* = 44 for pharmacotherapy, and *n* = 58 for a combined psychotherapy + pharmacotherapy treatment). An additional 150 persons (85 women and 65 men) without a depression diagnosis were recruited as a control group. All participants provided informed consent prior to their participation in the study. A summary of the sociodemographic characteristics of both groups is depicted in Table 1 together with value of *p*s for relevant statistical tests.

**Table 1:** Sociodemographic data for the group with a depression diagnosis and for the control group.

Variable		Depression diagnosis (*N* = 150)	Control group (*N* = 150)	value of *p*^a,b^
Gender	Male	44 (29.33%)	65 (43.33%)	0.016^a^
	Female	106 (70.67%)	85 (56.67%)	
Age	Mean	35.14	38.79	0.011^b^
	Standard dev	11.83	12.86	
	Range	18–63	18–66	
Place of residence	Village	23 (15.33%)	35 (23.33%)	0.408^a^
	City > 20.000	28 (18.67%)	31 (20.67%)	
	City > 50.000	16 (10.67%)	12 (8.00%)	
	City > 100.000	24 (16.00%)	21 (14.00%)	
	City > 200.000	59 (39.33%)	51 (34.00%)	
Education	Primary	8 (5.33%)	2 (1.33%)	0.114^a^
	Secondary	75 (50.00%)	85 (56.67%)	
	Higher	67 (44.00%)	63 (42.00%)	
Occupational	Full-time job	100 (66.67%)	97 (64.67%)	0.936^a^
status	Part-time job	18 (12.00%)	19 (12.67%)	
	Unemployed	32 (21.33%)	34 (22.67%)	

^a^*χ*^2^-test; ^b^*t*-test.

For place of residence, educational level, and occupational status no differences between samples were found (*p*s > 0.05), but the sample with a depression diagnosis had a significantly lower mean age (35.14) than the control group (38.79), *t*(298) = 2.56, *p* = 0.011, and a greater proportion of females to males (63.7% vs. 56.7 in the control group), *χ*^2^(1, *n* = 300) = 6.36, *p* = 0.016. We attended to the latter differences in subsequent analyses (ANCOVAs) but inclusion of age and/or gender as covariates did not alter any of the significance levels reported.

### Measures

All values of Cronbach’s alpha below were calculated for the current study.

#### Time perspective

Zimbardo Time Perspective Inventory (ZTPI, Zimbardo and Boyd, 1999, Polish adaptation by Cybulko and Zieliński) was used to measure time perspective. The inventory consists of 56 statements concerning time (e.g., ‘Happy memories of good times spring readily to mind’), rated on a 5-point Likert scale ranging from “very untrue” (coded as 1) to “very true” (coded as 5). The reliability rates of five dimensions in the present study were as follows: Past Negative (PN, *α* = 0.83), Past Positive (PP, *α* = 0.66), Present Hedonistic (PH, *α* = 0.82), Present Fatalistic (PF, *α* = 0.67), and Future (F, *α* = 0.80).

An aggregate score, reflecting differences from a proposed ideal constellation of ZTPI scores across all five subscales, referred to as Deviation from a Balanced Time Perspective (DBTP), was calculated in accord with the revised (DBTP-r) formula in Jankowski et al. (2020), that was found to improve on the original formula in terms of association of the scores with indicators of well-being:

$\mathrm{DBTP=}\;\sqrt{\begin{array}{l} {\left(1-ePN\right)}^{2}+{\left(5-ePP\right)}^{2}+{\left(1-ePF\right)}^{2}\\ +{\left(3.4-ePH\right)}^{2}+{\left(5-eF\right)}^{2} \end{array}}$

Expression with postscript e denotes the observed mean score for the individual (e.g., ePN = mean for the Past Negative subscale) and the first value is the proposed ideal mean value for that scale (e.g., PN = 1).

#### Cognitive fusion

Cognitive fusion was measured using the Cognitive Fusion Questionnaire (CFQ, Gillanders et al., 2014; Polish translation by Baran et al., 2019). CFQ consists of seven items (e.g., ‘I struggle with my thoughts’), rated on a 7-point Likert scale ranging from 1 (never true) to 7 (always true). In the present study, Cronbach’s alpha in the current sample (total group) was α = 0.94.

#### Self-compassion

The Self-Compassion Scale Short (Raes et al., 2011; Polish translation by Kocur et al., 2022) was used to measure self-compassion. The scale consists of 12 items (e.g., ‘When I’m going through a very hard time, I give myself the caring and tenderness I need’) rated on a 5-point scale ranging from 1 (almost never) to 5 (nearly always). In the present study, Cronbach’s alpha was *α* = 0.78.

#### Depressive symptoms

Depression, Anxiety and Stress Scale in the 21-item version (DASS-21, Lovibond and Lovibond, 1995, Polish translation by Makara-Studzińska et al., in preparation) was used to measure depressive symptoms. Items (e.g., *I felt life was meaningless*) are rated on a four-point Likert scale ranging from 0 “rarely or never” (coded as 0) to “most of the time” (coded as 3). Due to the scope of the current study, only the items belonging to the depression scale was used. Cronbach’s α for this scale was 0.94.

#### Statistical methods

Simple associations of manifested scores were evaluated using Pearson correlations. Group differences were examined using independent *t*-tests, and, to control for current symptom level, Analyses of Covariance (ANCOVA). The *α*-level was set to 0.05. Additionally, we computed Cohen’s *d* as a measure of effect size, using common cutoff values 0.80 (large), 0.50 (medium), and 0.20 (small) to grade the size of the effect (Cohen, 1988).

Structural equation modeling (SEM) was employed using IBM SPSS AMOS 28 to test a model involving cognitive fusion and self-compassion as latent-level predictors of depressive symptoms including DBTP as a mediator. Potential group differences were first attended to, imposing equality constraints on item loadings and structural weights to see if such constraints resulted in poorer model fit. We used a parceling approach to reduce the number of observed variables in the model. This should result in a more parsimonious model while providing increased power to test the relations among latent variables (Little et al., 2013). Consistent with prior studies (Joeng and Turner, 2015; Pyszkowska and Rönnlund, 2021) the average score for self-kindness and the reverse scored self-judgment (SC1), common humanity and the reverse scored isolation (SC2), and mindfulness and the reverse scored over identification (SC3) were used as three indicators of a self-compassion construct. Cognitive fusion as reflected by the CFQ is a unitary construct. Hence three item parcels (CF1 for the average of items 1, 6, 7; PF2 for items 2, 5; and PF3 for items 1, 3) were created using random assignment (Matsunaga, 2008). Model fit was evaluated using the following indexes: Comparative Fit Index (CFI), where values >0.95 are considered to indicate good model fit, and Squared Error of Approximation, RMSEA, where values <0.06 are considered to indicate good model fit and values of 0.08 or lower were taken to indicate acceptable model fit (Hu and Bentler, 1999).

## Results

We examined mean level differences of the time perspective measures and other measures (depressive symptoms, cognitive fusion, self-compassion) between the depression group and the control group. In the first step item distributions and the assumption of homogeneity of variance were examined, and for none of the variables, skewness or kurtosis exceeded common cut-off values (<1.0 for both parameters across all variables). Levene’s test indicated no need for adjustment for non-equal variance except for depressive symptoms and the measure of fusion. The means (*M*), standard deviation (*SD*), results of independent sample *t*-tests, and ANCOVAs controlling for depressive symptoms, are presented in Table 2.

**Table 2:** Descriptive data for the control (no depression) and clinical (depression diagnosis) group together with results of independent sample *t*-test, effect sizes (Cohen’s *d*), and results from ANCOVAs controlling for depressive symptoms.

	No depression			Depression diagnosis			*t*-value	Value of *p*	Cohen’s *d*	*F*-value	*MSe*	*p*-value
	M	SD	SE	M	SD	SE						
DBTP	4.12	0.75	0.06	4.69	0.76	0.06	−6.53	< 0.001	−0.075	6.48	0.47	0.011
Past negative	2.89	0.74	0.06	3.34	0.66	0.05	−5.56	< 0.001	−0.64	1.12	0.37	0.290
Past positive	3.21	0.69	0.06	3.09	0.74	0.06	1.45	0.144	0.17	2.14	0.51	0.144
Present fatalistic	2.94	0.70	0.06	3.40	0.77	0.06	−5.44	< 0.001	−0.63	2.05	0.44	0.153
Present hedonistic	3.05	0.67	0.05	3.30	0.78	0.06	−2.91	0.004	−0.33	0.48	0.44	0.490
Future	3.34	0.51	0.04	3.32	0.55	0.04	0.60	0.720	0 0.04	0.94	0.28	0.332
Depressive symptoms	17.08	10.83	0.88	27.34	8.69	0.71	−9.05	< 0.001	−1.04	–	–	–
Cognitive fusion	27.24	10.33	0.84	36.83	7.72	0.63	−9.11	<0 0.001	−1.05	11.72	42.4	<0.001
Self-compassion	36.24	7.73	0.63	30.79	7.05	0.58	6.37	< 0.001	0.73	10.13	49.02	0.002

The results showed significant mean-level group differences across most of the measures. The depression group exhibited significantly greater DBTP scores (*t* = −6.53, *p* < 0.001), the TP measure yielding the largest effect size (*d* = −0.75), but also higher means on the Past Negative (*t* = −5.56, *p* < 0.001), Present Hedonistic (*t* = −2.91, *p* = 0.004), Present Fatalistic (*t* = −5.44, *p* < 0.001) ZTPI dimensions. As expected, there was additionally a significant difference for depressive symptoms (*t* = −9.05, *p* < 0.001) and cognitive fusion (*t* = −9.11, *p* < 0.001), with higher means for the depression group, but lower mean for the measure of self-compassion (*t* = 6.37, *p* < 0.001). The groups did not differ on Future TP and Past Positive TP.

As we can see, the *F*-values (and corresponding value of *p*s) for the ANCOVAs, indicate that for the three variables considered as predictors of depressive symptoms in subsequent analyses, namely DBTP [*F*(1, 297) = 3.02, *p* = 0.011], cognitive fusion [*F*(1, 297) = 11.73, *p* < 0.001] as well as self-compassion [*F*(1, 297) = 10.13, *p* < 0.01], the group difference remained statistically significant, whereas differences for individual ZTPI scales did not.

Next, we investigated the associations between the main study variables (time perspective cognitive fusion, self-compassion, and depressive symptoms). Table 3 presents the descriptive statistics (*M, SD*) and bivariate associations (Pearson *r*s) of the variables for the entire sample (*N* = 300).

**Table 3:** Descriptive statistics (*M, SD*) and bivariate correlations (Pearson’s *r*) of the study variables.

	M	SD	1	2	3	4	5	6	7	8
1. DBTP	4.41	0.81	—							
2. Past negative TP	3.12	0.73	0.72***	—						
3. Past positive TP	3.15	0.72	−0.38***	0.01	—					
4. Present fatalistic TP	3.17	0.77	0.69***	0.67***	0.25***	—				
5. Present hedonistic TP	3.18	0.73	0.34***	0.54***	0.47***	0.72***	—			
6. Future	3.33	0.53	−0.11	0.33***	0.37***	0.25***	0.28***	—		
7. Cognitive fusion	32.04	10.30	0.56***	0.63***	−0.07	0.50***	0.38***	0.10	—	
8. Self-compassion	33.52	7.88	−0.41***	−0.37***	0.33***	−0.15*	0.07	0.11	−0.58***	—
9. Depressive symptoms	22.21	11.07	0.52***	0.56***	−0.02	0.51***	0.43***	0.06	0.77***	−0.43***

*p < 0.05, **p < 0.01, ***p < 0.001.

As we can see, DBTP showed significant correlations with depressive symptoms (*r* = 0.52), cognitive fusion (*r* = 0.56), and self-compassion (*r* = −0.41). Cognitive fusion exhibited significant relationships with Past Negative (*r* = 0.63), Present Hedonistic TP (*r* = 0.38), and Present Fatalistic (*r* = 0.50). Similar results were obtained with depressive symptoms which showed significant associations with cognitive fusion (*r* = 0.77). Self-compassion was significantly negatively correlated with Past Negative (*r* = −0.37), Present Fatalistic (*r* = −0.15) depressive symptoms (*r* = −0.43), and cognitive fusion (*r* = −0.57), and positively with Past Positive (*r* = 0.33).

Finally, as outlined in the introduction, we tested a hypothetical model including cognitive fusion and self-compassion as predictors of depressive symptoms, directly or via DBTP (i.e., as a mediator). Initial multi-group analyses indicated that imposing equality constraints for the depressed and non-depressed subsamples with regard to measurement weights and structural weights did not significantly worsen model fit (ΔCFI <0.01, *p* > 0.05), suggesting that the basic relations of the constructs were similar regardless of the depression/no depression distinction. Consequently, the combined sample was used to test the structural model.

**Figure 1:**
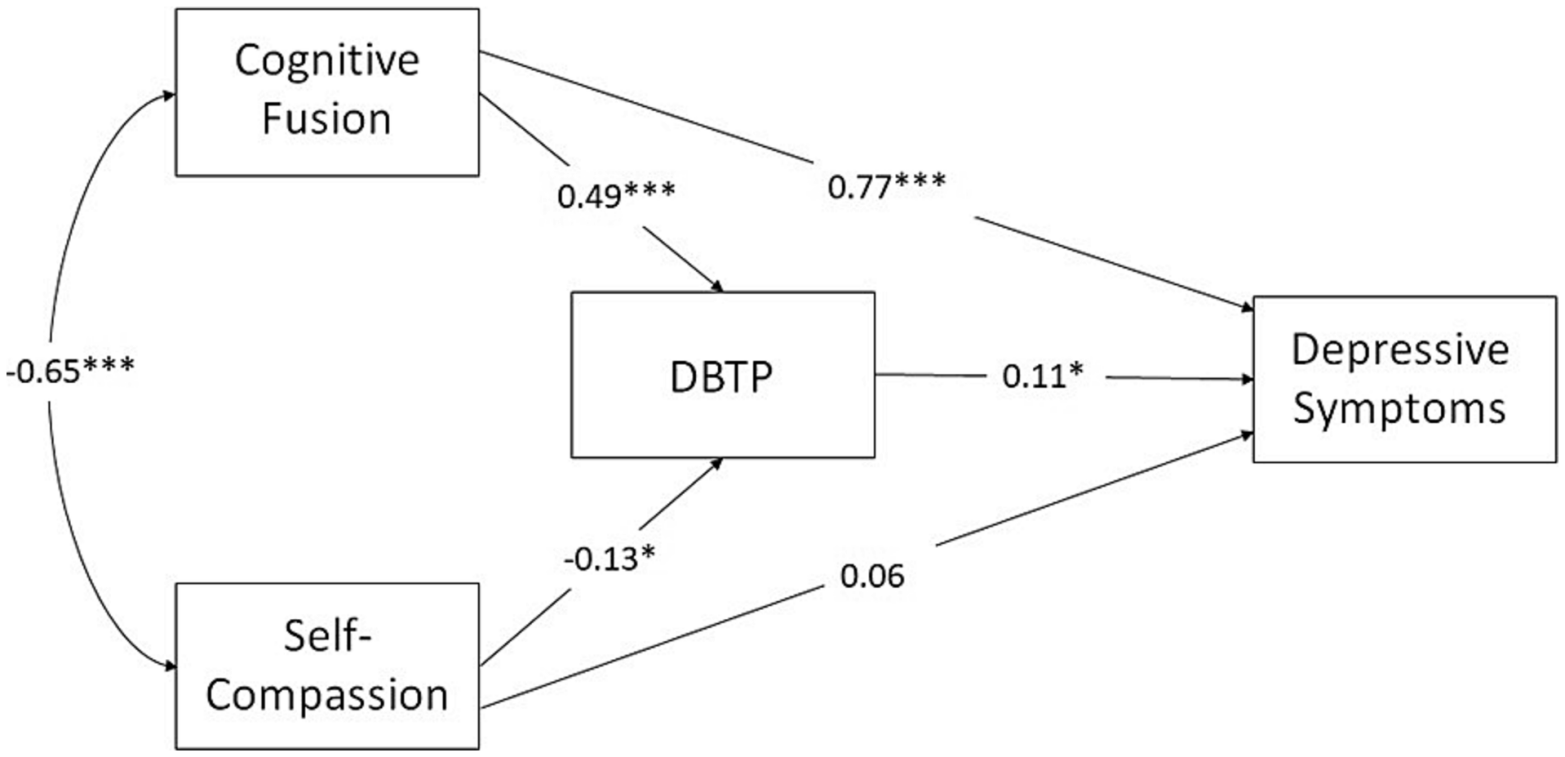
Summarizes the model and the results. The model showed good fit as judged the fit indices (CFI = 0.99, RMSEA = 0.019). The cognitive fusion and self-compassion factors were negatively associated (*r* = −0.65). Moreover, cognitive fusion was a significant (direct) predictor of depression (*c*’ = 0.77) and of DBTP (*β* = 0.49), whereas the paths from self-compassion to depression (0.06) and DBTP (−0.13, *p* = 0.08) were not statistically significant. Finally, the path from DBTP to depression was significant (*β* = 0.11, *p* < 0.05), indicating that, regardless of other factors, larger deviations from the balanced time perspective were associated with higher symptom levels.

Figure 1 summarizes the model and the results. The model showed good fit as judged the fit indices (CFI = 0.99, RMSEA = 0.019). The cognitive fusion and self-compassion factors were negatively associated (*r* = −0.65). Moreover, cognitive fusion was a significant (direct) predictor of depression (*c*’ = 0.77) and of DBTP (*β* = 0.49), whereas the paths from self-compassion to depression (0.06) and from self-compassion to DBTP (−0.13, *p* = 0.08) were not statistically significant. Finally, the path from DBTP to depression was significant (*β* = 0.11, *p* < 0.05), indicating that, regardless of other factors, larger deviations from the balanced time perspective were associated with higher symptom levels.

The standardized total effect of cognitive fusion on symptoms (*c* = 0.82) was highly significant (95% BCI: 0.73–0.91, *p* < 0.001, two-tailed) with a non-significant total effect (0.05, 95% BCI: −0.06-0.16) in the case of self-compassion. Evidence of a significant indirect effect on depressive symptoms via DBTP was obtained for cognitive fusion (0.06, 95% BCI; 0.01–0.11, *p* < 0.05) even though the estimate of the direct effect of *CF* on depressive symptoms (*β* = 0.77, 95% BCI: 0.66–0.86) was not significantly different from the total effect (0.82). In total, the predictor variables together accounted for 63% of the variance in depressive symptoms.

## Discussion

The objectives of the study were to compare time perspective profiles, including DBTP, and levels of cognitive fusion, self-compassion and DBTP in a sample of individuals with a depression diagnosis and a control group and to explore the mediating role of DBTP in the relationship between cognitive fusion, self-compassion, and depressive symptoms.

As expected, the clinical sample showed significantly higher levels of cognitive fusion (Bardeen and Fergus, 2016) and lower self-compassion (Krieger et al., 2013) compared with the sample with no depression diagnosis. Of primary concern, the DBTP measure differed substantially for the depression group compared to the control group. One may also note that the effect size for the DBTP score was larger than for the individual TP scales, suggesting that depression may be characterized bias across facets of TP that sum up to a difference (*d* = 0.75) close to common cutoff for a large effect (0.8). The current study seems to suggest a considerably stronger relationship between depression and DBTP as compared to the extant studies (McKay and Cole, 2020; McKay and Cole, 2023), also when correlations of DBTP and depressive symptoms are considered as continuous variables. In any case, the fact that the group difference in DBTP, unlike separate ZTPI subscales, remained significant even when current symptom level was controlled for substantiates the DBTP-depression association. The latter result in additions suggests that DBTP may act a vulnerability factor to develop depression.

Consistent with prior studies, the depression group furthermore showed higher levels of Past Negative and Present Fatalistic, with no differences for Future and Past Positive. Additionally, the depression group exhibited significantly higher rates of Present Hedonistic, which is in line with scarce prior research (Davies and Filippopoulos, 2015; McKay et al., 2016) but at odds with the study by Lefèvre et al. (2019). It can be hypothesized that a Present Hedonistic TP is related not only to pleasure seeking but also committing to maladaptive coping strategies, especially emotion-oriented or distraction-seeking (Bodecka et al., 2021). As persons with depression experience elevated rates of anhedonia (Zhang et al., 2016), individuals with higher levels of Present Hedonistic may be unable to fulfill their needs for stimulation due to the blockage of pleasant stimuli, and that would enhance depressive mood (Bodecka et al., 2021). Of note, as no significant relationships were obtained between Present Hedonistic and self-compassion in the current study, it can be assumed that a present hedonistic time perspective is not associated with self-care or reducing one’s suffering.

As concerns association between the major variables, depressive symptoms were significantly associated with cognitive fusion (Ranjbar et al., 2022), DBTP, Past Negative, Present Fatalistic, and Present Hedonistic TPs (Lefèvre et al., 2019), and showed a significant negative association with Past Positive (Anagnostopoulos and Griva, 2012), in line with prior studies, with self-compassion exhibiting negative relationships with both depressive symptoms (Carvalho et al., 2019) and cognitive fusion (Böge et al., 2022).

The proposed models of the relations between variables showed good fit, and despite variations in mean levels, no difference was found for the depression/no depression samples. Thus, according to these models, cognitive fusion acted as a main predictor of depressive symptoms across samples, with a weaker significance of DBTP, and an insignificance of self-compassion. That is in line with previous research suggesting that the dominance of negative thought patterns (including time frames) and the entanglement with cognitive symptoms of depression (e.g., rumination, narrow negative perspective-taking) cause the elevated depressive symptoms (Gillanders et al., 2014) and diminish the role of self-compassion (Pyszkowska and Rönnlund, 2021). The current findings are partially in line with Ranjbar et al. (2022) who demonstrated significant links between DBTP, cognitive fusion, and depression in a general population, although in case of the current study, this co-occurrence of DBTP and cognitive fusion in predicting depression was slightly stronger in a general (control) population. It could suggest that perceiving thoughts as irrevocable facts, a core mechanism of cognitive fusion, is of greater importance in developing depressive symptoms than solely a negative focus on a particular time frame, being in line with a complex psychopathology of depression. As individuals with depression exhibit higher rates of fusion when compared to a control group, they display a greater entanglement with distressing thoughts regardless of their content. Hence, it is not the presence of negatively biased temporal thinking *per se* that causes the individual’s suffering, but rather the entanglement with, and belief in these thoughts. Further studies in clinical samples are required in order to develop a better understanding of the relationships in question.

Self-compassion proved to be insignificant in predicting depressive symptoms in both samples, being outweighed by cognitive fusion and DBTP (in a general sample). As proposed by Ranjbar et al. (2022), time-entrapped fused thoughts may elicit depressive symptoms associated with both negative views of the past (e.g., ruminations, regret, shame), and the future (e.g., catastrophic worry), preventing the compassionate thoughts toward oneself from occurring. That could be considered as a logical consequence of cognitive depressive symptoms as “the bad supersedes the good,” a cognitive bias aimed at avoiding thought content that does not conform to the well-known negative pattern (Beshai et al., 2016). These results are partially in line with Gillanders et al. (2014) who demonstrated self-compassion’s insignificance in predicting nor moderating depressive symptoms, with cognitive fusion and avoidant coping strategies being main predictors in a heterogenous sample of former cancer patients. On the other hand, Carvalho et al. (2019) suggest that self-compassion might moderate between cognitive fusion and depressive symptoms, with higher rates of self-compassion reducing the fusion – further studies in this area are required as the current data is inconclusive.

The findings of the current study have significant clinical implications. First, DBTP and cognitive fusion are of significance for the occurrence of depressive cognitive symptoms in both clinical and non-clinical samples hence the focus on developing flexible and adaptive ways of thinking should be aimed as specific a therapeutic goal among persons exhibiting such symptoms. Also, consistent with Hayes and Hofmann’s (2020) or Neff’s (2003) views on the universality of human’s suffering and the limiting function of the labels of mental disorders, the results highlight that cognitive fusion, an unbalanced time perspective and/or negativity-focused time perspectives are of significant importance for the occurrence of depressive symptoms, regardless of the nosological diagnosis. In turn, focusing on developing competences aimed at defusion, balance and flexibility are universal and may be beneficial in any patient group as depressive symptoms may occur in various contexts and disorders. Additionally, as cognitive fusion and DBTP were of greater significance for the occurrence of depressive symptoms than self-compassion, it can be suggested to first develop basic skills regarding defusion and perspective-taking, and then focus on one’s self-kindness. Reducing cognitive fusion, fostering more balanced approach to ones experiences, and increasing self-compassionate attitudes toward oneself may be developed through Acceptance and Commitment Therapy (ACT), or Compassion-Focused Therapy (CFT).

### Strengths, limitations, and future directions

The current study was to our knowledge the first to date to compared DBTP for a group with clinical depression and a non-clinical sample. It adds to an existing body of literature regarding depressive symptoms and DBTP, as well as associations between cognitive fusion and rigid cognitive schemas. Despite its strengths, such as the inclusion of a clinical and relatively large sample, and the use of sophisticated analytical techniques, it clearly has limitations. First, the participants were recruited via a research panel which means that self-selection factors could potentially have an effect. Second, the data consisted of self-reports which and could hence be susceptible to common-method biases. Third, depression symptoms were measured using DASS-21 as a one-factor variable, which disallowed for the distinguishment of multi-faceted aspects of depression (e.g., ruminations, morbid thoughts, negative self-image, chronic exhaustion, executive dysfunction, memory or concentration impairment, etc.) and its links to the variables studied. Third, the original version of the ZTPI used in the current study involves a single (mainly positive) future dimension. However, Åström et al. (2019a,b), for example, demonstrated a significant link between a separate Future Negative subscale and depressive symptoms. Thus, inclusion of such a scale and incorporating as part of the DBTP measure (Rönnlund et al., 2018) should be expected to give an even stronger differentiation of clinical/non-clinical samples. Finally, the present study was cross-sectional which precludes casual inferences.

To examine of the suggested causal links in the model. Future research should be focused on longitudinal observations and prospective designs regarding the dynamics between variables, especially the relative merits of cognitive fusion, time perspective, in predicting future depressive symptoms or as risk factors for developing development depression. Given that cognitive fusion is one of the main issues in Acceptance and Commitment Therapy, it should be observed how therapeutic ACT interventions aimed at defusion would reduce biases of the individual’s time perspective profile and thereby achieve a more balanced time perspective.

## Data availability statement

The raw data supporting the conclusions of this article will be made available by the authors, without undue reservation.

## Ethics statement

The studies involving humans were approved by the University of Silesia’s Ethics Committee. The studies were conducted in accordance with the local legislation and institutional requirements. The participants provided their written informed consent to participate in this study.

## Author contributions

AP: Conceptualization, Formal analysis, Funding acquisition, Investigation, Methodology, Project administration, Writing – original draft. EÅ: Conceptualization, Methodology, Validation, Writing – review & editing. MR: Conceptualization, Data curation, Formal analysis, Methodology, Writing – review & editing.
